# Supplementary material for: Dielectric Responses of Cytosolic Water Change with Aging of Circulating Red Blood Cells
Source: Cells. 2025 Mar 24;14(7):486. doi: 10.3390/cells14070486 (PMC11987982; doi:10.3390/cells14070486)
Supplement: Supplementary file 1 [file cells-14-00486-s001.zip › cells-3456970-supplementary.pdf]

Dielectric Responses of Cytosolic Water Change with Aging of Circulating Red Blood Cells

Larisa Latypova, Cindy Galindo, Leonid Livshits, Rodolfo Victor Teope, Dan Arbell, Gregory Barshtein, Anna Bogdanova and Yuri Feldman

RBC fractionation by Percoll density gradient.

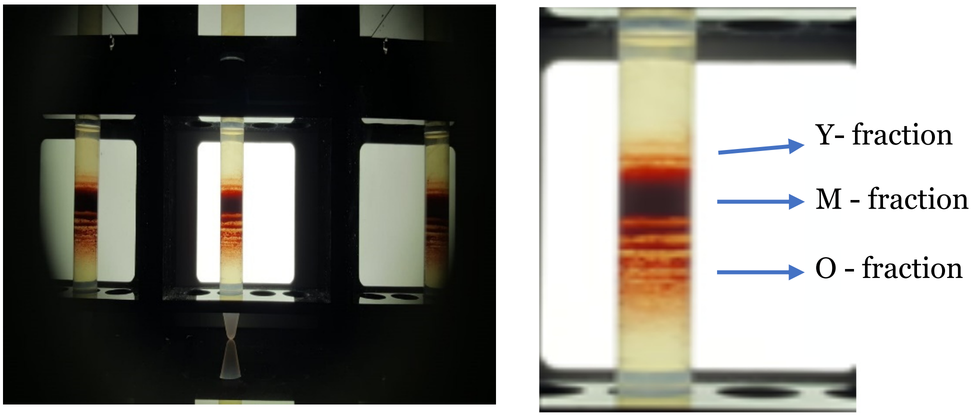

Figure S1. Left Panel - RBC sample (from Donor 1) separated to their density by a standard Percoll gradient. Right panel - The light fraction (Y-fraction) is at the top of the tube (makes up 5.31% of the total weight); the most abundant (62.16 %) fraction (M-fraction) is in the middle of the tube; the heaviest fraction (O-fraction) is on the bottom of the tube (makes up 28.68% of the total weight).

Does the separation of RBCs on Percoll cause alteration of cell features?

The control experiment was designed to determine whether the properties of red blood cells (RBCs) change after they are separated into fractions. To achieve this, we performed additional experiments comparing the characteristics of native RBCs with those that underwent Percoll processing. This comparison revealed no changes in hematological indices (Table S1) and the dielectric properties of intracellular water (Fig. S1 and Table S2). The deformability of erythrocytes also remained unchanged (Table S3). These results enabled us to conclude that the process of RBC separation using Percoll does not alter cell characteristics, allowing us to use this method to separate cells into fractions (subpopulations) based on cell density.

<u>*Hematological indices of separated RBCs*</u>

Table S1. Hematological indices for control samples (Native *vs*. Percoll control).

Parameters	Percoll Control		Native
	Mean ± SE	*Vs* Native, p value	Mean ± SE
MCHC, g/dl	33.1±0.3	NS	32.8±0.4
MCV, fL	85.5±1.2	NS	86.3±1.4
MCH, pg	28.5±0.4	NS	28.0±0.4

The data presented in **Table S1** shows that separation on Percoll allowed us to obtain RBC fractions with significantly different hematological indices (MCHC, MCV, MCH).

Dielectric properties of cytoplasmic water

Figure S1 and **Table S2** show the fitting parameters (Δε; τ and α) obtained after using a mixture formula Kraszewski (**Eq. 2**) for the control experiment.

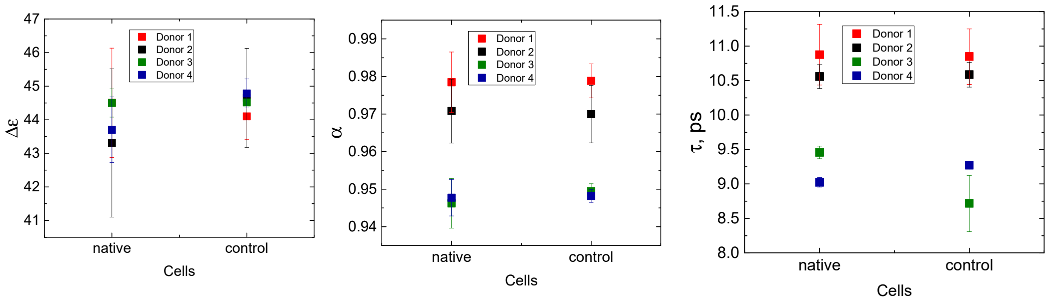

Figure S2. Fitting parameters of the control experiment for four healthy donors.

As it can be seen from (**Figure S1)**, fitting parameters for these 2 cases are the same. Comparison of the results obtained for the two groups shows that their difference is not significant (**Table S2**).

Table S2. Fitting parameters for control samples (**Native *vs*. Percoll control**).

Parameters	Percoll Control		Native
	Mean ± SE	*Vs* Native, p value	Mean ± SE
Δε	44.5±0.1	NS	44.0±0.3
α	0.962±0.008	NS	0.961±0.007
τ, ps	9.86±0.44	NS	9.98±0.38

Deformability of separated RBCs

The analysis of the deformability of the RBCs also showed that cells functionality does not change after Percoll treatment, and as follows from **Table S3**.

Table S3. RBC deformability features for control samples (Native vs. Percoll control).

Parameters	Percoll Control		Native
	Mean ± SE	*Vs* Native, p-value	Mean ± SE
MER	1.32±0.02	NS	1.33±0.02
%, UDFC	11.1±1.2	NS	10.9±1.4

Conclusion:

In this section, we compared properties of RBCs before and after separation by Percoll gradient. For this purpose, we used methods sensitive to cell hydration. Unlike van Cromvoirt *et al.* [5], who postulated that *fractionation was associated with overhydration of RBC*, we did not find

any changes in the state of erythrocytes. In our case, separation by Percoll gradient was not accompanied by a change of parameters that expressed the RBC hydration state.

Does the separation of RBCs on Percoll cause alteration of cell features?

Table S4. Hematological indices for three RBCs subpopulations.

Parameters	Native		O-fraction		M-fraction		Y-fraction
	Mean ± SE	Vs Y, p value	Mean ± SE	*Vs* Y, p value	Mean ± SE	*Vs* Y, p-value	Mean ± SE
MCHC, g/dl	32.8±0.4	*0.002*	35.0±0.4	*0.0001*	32.5±0.4	*0.0012*	28.9±0.5
MCV, fL	86.3±1.5	*0.0025*	81.4±1.6	*0.0002*	86.6±1.4	*0.0015*	94.7±1.5
RDW-SD, fL	38.2±1.5	*0.033*	34.4.4±1.3	*0.003*	37.0±1.3	*0.014*	44.4±1.5
MCH, pg	28.0±0.57	*NS*	28.5±0.74	*NS*	28.1±0.41	*NS*	27.4±0.52

Table S5. Fitting parameters for O, M, and Y-fractions of RBCs for four healthy donors.

**Parameters**	Native		O-fraction		M-fraction		Y-fraction
	Mean ± SE	Vs Y,p value	Mean ± SE	*Vs* Y, p value	Mean ± SE	,*Vs* Y p value	Mean ± SE
Δε	44.0±0.3	*0.008*	43.5±1.2	*0.015*	46.6±1.2	*0.014*	49.9±1.7
α	0.961±0.007	*NS*	0.957±0.008	*0.008*	0.960±0.007	*0.01*	0.965±0.013
τ, psec	9.98±0.38	*NS*	10.2±0.54	*0.039*	10.1±0.44	*0.02*	9.04±0.92

Table S6. RBCs deformability features for native RBCs and their fraction (L, M, and H-fractions of RBCs).

Parameters	Native		O-fraction		M-fraction		Y-fraction
	Mean ± SE	*Vs*. L, p-value	Mean ± SE	*Vs*. L, p-value	Mean ± SE	*Vs*. L, p-value	Mean ± SE
MER	1.33±0.02	0.01	1.30±0.1	0.0004	1.34±0.02	0.006	1.40±0.02
%, UDFC	10.9±1.4	0.02	12.7±1.1	0.0002	10.3±0.8	0.007	7.7±0.9

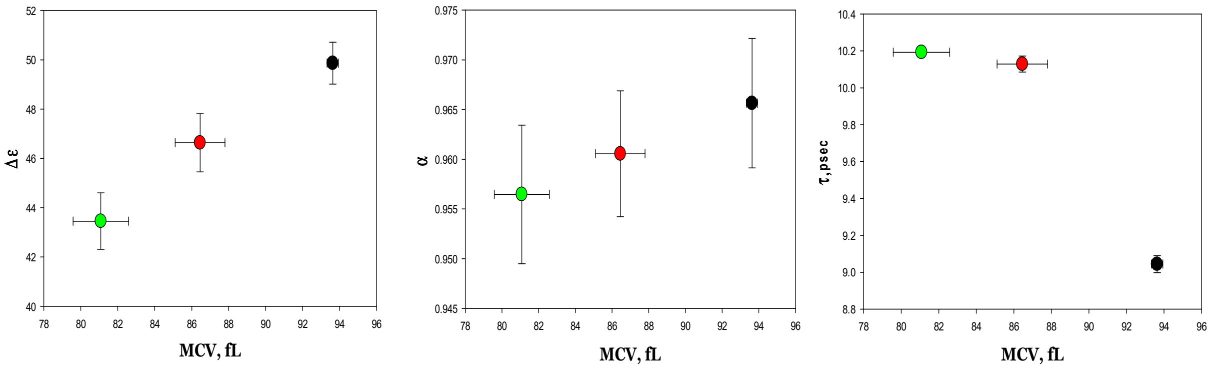

Figure. S3. Correlation between dielectric fitting parameters and MCV for three RBCs subpopulations; Y (black), M (red), and O - fraction (green). Each date presented as Mean ± SE from four samples.

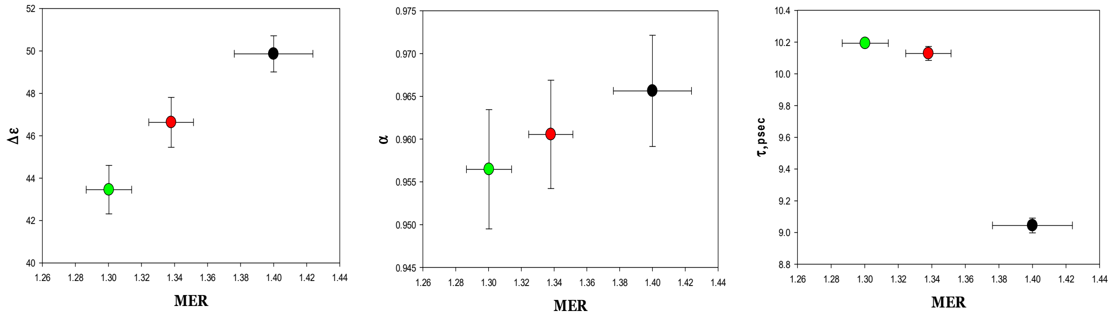

Figure S4. Correlation between dielectric fitting parameters and MER for three RBCs subpopulations; light (black), medium (red), and high-density fraction (green). Each date presented as Mean ± SE from four samples.
